# Supplementary material for: A covalent chemical probe for Chikungunya nsP2 cysteine protease with antialphaviral activity and proteome-wide selectivity
Source: Sci Rep. 2025 Mar 1;15:7264. doi: 10.1038/s41598-025-91673-x (PMC11873239; doi:10.1038/s41598-025-91673-x)

## Supporting Information

# A Covalent Chemical Probe for *Chikungunya* nsP2 Cysteine Protease with Antialphaviral Activity and Proteome-wide Selectivity

Anirban Ghoshal[1,2], Edwin G. Tse[1,2], Mohammad Anwar Hossain[1,2], Kesatebrhan Haile Asressu[1,2], Eric M. Merten[2,3], John D. Sears[2,4], Stefanie Howell[1,2], Sumera Perveen[2,5], Jane Burdick[2,6], Noah L. Morales[2,6], Sabian A. Martinez[2,6], Isabella Law[2,6], Bennett J. Davenport[2,7], Thomas E. Morrison[2,7], Zachary J. Streblow[2,8], Daniel N. Streblow[2,8], Angie L. Mordant[9], Thomas S. Webb[9], Aurora Cabrera[9], Laura E. Herring[9], Cheryl H. Arrowsmith[2,5], Kenneth H. Pearce[2,3], Nathaniel J. Moorman[2,4], Mark T. Heise[2,4,6], Rafael M. Couñago[1,2,10], Peter J. Brown[1,2], and Timothy M. Willson[1,2,*]

[1]Structural Genomics Consortium, UNC Eshelman School of Pharmacy, University of North Carolina at Chapel Hill, Chapel Hill, NC 27599, USA.
[2]READDI AViDD Center, University of North Carolina at Chapel Hill, Chapel Hill, NC 27599, USA.
[3]UNC Eshelman School of Pharmacy, Center for Integrative Chemical Biology and Drug Discovery, University of North Carolina at Chapel Hill, Chapel Hill, NC 27599, USA
[4]Department of Microbiology and Immunology, University of North Carolina at Chapel Hill, Chapel Hill, NC, 27599, USA
[5]Structural Genomics Consortium, University of Toronto, Toronto, Ontario, M5G 1L7, Canada
[6]Department of Genetics, University of North Carolina at Chapel Hill, Chapel Hill, NC, 27599, USA
[7]Department of Immunology and Microbiology, University of Colorado School of Medicine, Aurora, CO 80045, USA.
[8]Vaccine & Gene Therapy Institute, Oregon Health & Science University, Beaverton, OR, 97006, USA
[9]UNC Metabolomics and Proteomics Core, University of North Carolina at Chapel Hill, Chapel Hill, NC, 27599, USA
[10]Center of Medicinal Chemistry, Center for Molecular Biology and Genetic Engineering, University of Campinas, 13083-886-Campinas, SP, Brazil

*Correspondence: tim.willson@unc.edu


| | Table of Contents | Page |
|---|---|---|
| Table S1 | Inhibitory effects of **3** on human and viral cysteine proteases | S2 |
| Figure S1 | Chemical stability of **3** and **4** at room temperature | S3 |
| Figure S2 | DLS data for **3** and **4** | S4 |
| Figure S3 | GSH stability of **3** | S5 |
| Figure S4 | Dose response of **3** on the EEEV-nLuc replicon assay | S6 |
| Figure S5 | Cell toxicity data upon 48 h exposure of **3** and **4** | S6 |
| Figures S6 | Site of covalent modification of CHIKV nsP2pro | S7 |
| Figure S7 | Dose response curves of **VS** | S8 |
| Figure S8 | TAMRA fluorescence chemoproteomics of **VSS** | S9 |
| Figure S9 | Structure of MOM-**1** | S10 |
| Figures S10–S21 | Analytical data for new compounds | S11–S16 |
| Figure S22 | Uncropped images of gels from Figure 4a and Figure 5b | S17 |

**Table S1**. Inhibitory effects of **3** on human and viral cysteine proteases

| Enzyme | Substrate | Control | % Inhibition | | | |
|---|---|---|---|---|---|---|
| | | | **3** (10 μM) | **Reference** (0.1 x $IC_{50}$)[a] | **Reference** (1 x $IC_{50}$)[a] | **Reference** (10 x $IC_{50}$)[a] |
| Cathepsin B | 5 μM Z-Leu-Arg-AMC | E-64 | 10 | 13 | 57 | 97 |
| Cathepsin F | 10 μM Z-Phe-Arg-AMC | Cystatin C | 10 | 12 | 50 | 88 |
| Cathepsin K | 70 μM Cathepsin K Substrate | E-64 | 3 | 12 | 46 | 99 |
| Cathepsin L | 5 μM Z-Leu-Arg-AMC | E-64 | 12 | 7 | 51 | 97 |
| Cathepsin S | 5 μM Z-Leu-Arg-AMC | E-64 | 14 | 14 | 55 | 91 |
| Cathepsin V | 5 μM Z-Leu-Arg-AMC | E-64 | 7 | 7 | 50 | 99 |
| MALT1 | 20 μM Ac-LRSR-AMC | Z-VRPR-FMK | 7 | 11 | 54 | 89 |
| A20 | 100 nM Ub-AMC | Ub-Aldehyde | 0 | 3 | 35 | 83 |
| Ataxin-3 | 100 nM Ub-AMC | Ub-Aldehyde | 7 | 8 | 47 | 90 |
| OTUD6B | 100 nM Ub-AMC | Ub-Aldehyde | 1 | 7 | 44 | 94 |
| UCHL1 | 100 nM Ub-AMC | Ub-Aldehyde | 3 | 2 | 30 | 97 |
| UCHL3 | 100 nM Ub-AMC | Ub-Aldehyde | 8 | 8 | 44 | 99 |
| USP1 | 100 nM Ub-AMC | Ub-Aldehyde | 1 | 19 | 73 | 96 |
| USP2 | 100 nM Ub-AMC | Ub-Aldehyde | 0 | 13 | 51 | 90 |
| USP5 | 100 nM Ub-AMC | Ub-Aldehyde | 1 | 13 | 55 | 97 |
| USP7 | 100 nM Ub-AMC | Ub-Aldehyde | 5 | 14 | 41 | 94 |
| USP8 | 100 nM Ub-AMC | Ub-Aldehyde | 5 | 18 | 53 | 89 |
| USP10 | 100 nM Ub-AMC | Ub-Aldehyde | 1 | 18 | 40 | 83 |
| USP14 | 100 nM Ub-AMC | Ub-Aldehyde | 8 | 11 | 46 | 95 |
| USP20 | 100 nM Ub-AMC | Ub-Aldehyde | 1 | 28 | 64 | 96 |
| 3CL Pro (MERS-CoV) | 10 μM 3CL MERS-Cov Substrate | Protease Cocktail | 1 | 6 | 42 | 83 |
| Papain-like Pro (SARS-CoV-2) | 25 μM PLPro Substrate | GRL0617 | 0 | 8 | 45 | 82 |
| 3CL Pro (SARS-CoV-2) | 50 μM 3CL Protease Substrate | GC376 | 5 | 8 | 48 | 92 |

[a]$IC_{50}$ of the control nsP2 protease inhibitor.

**Figure S1**. Chemical stability of **3** and **4** at room temperature

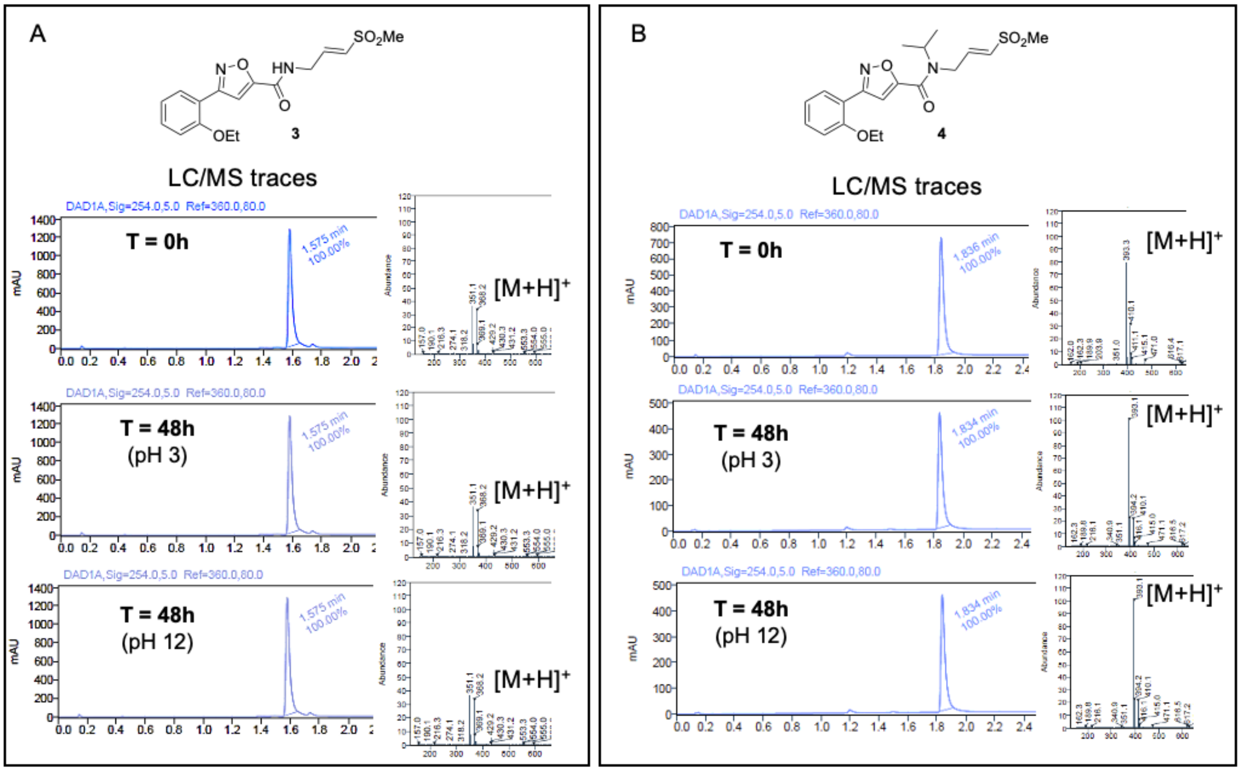

**Figure S2**. DLS data for **3** and **4**

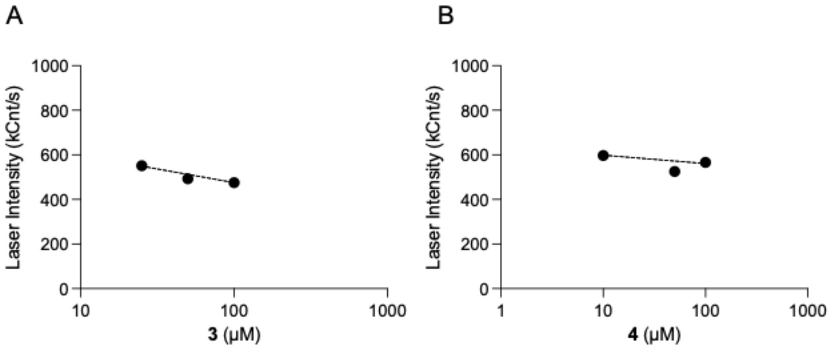


.

**Dynamic Light Scattering (DLS) Assay**. Aggregation behavior of chemical probe **3** and negative control **4** were determined by DLS in duplicate at 25 ºC in 25 mM HEPES buffer (pH 7.4) containing 0.003% Tween-20 and 1 mM DTT, using a 10 mM stock solution containing 2% DMSO. Light scattering was measured using a DynaPro Plate Reader III. Buffer with 2% DMSO control produced an average laser intensity of 858 kCnts/s.

**Figure S3.** GSH stability of **3**

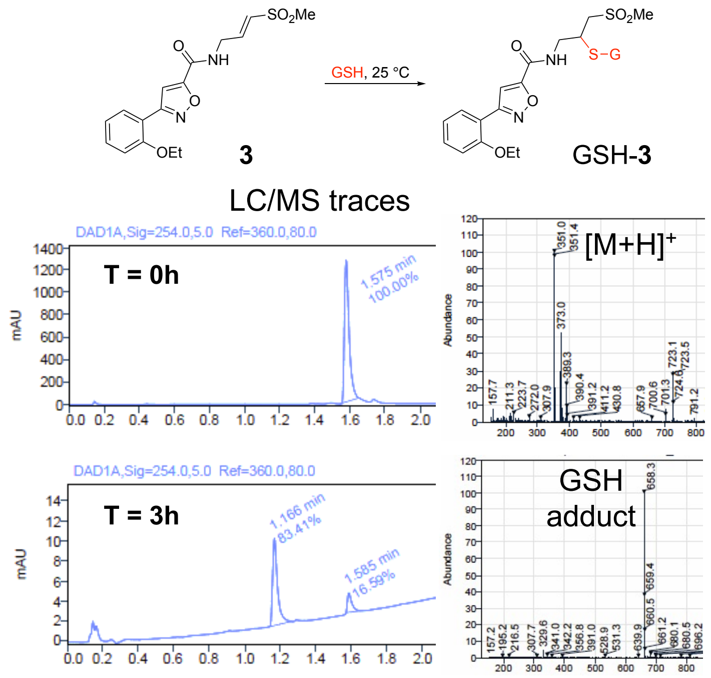


**Method for GSH stability.** A 10 mM solution of GSH (Sigma Aldrich Cat# G4251 (St. Louis, MO, USA)) was prepared in a pH 7.4 phosphate buffer. A 10 mM DMSO solution of Compound **3** was diluted in phosphate buffer to give a solution at 100 µM with 1% DMSO. At time zero (t = 0), 50 µL of the 100 µM test compound solution was added to an Eppendorf tube containing 50 µL phosphate buffer and 50 µL of 10 mM GSH solution. The final concentrations of the compound and GSH were maintained at 50 µM and 5 mM, respectively. The Eppendorf tube was vortexed, and then the sample was transferred to a high-recovery autosampler vial for LCMS analysis. Analysis was performed at 0, 1, and 3 h time points, and the percentage of GSH adduct formation was calculated using Agilent LCMS software (OpenLab CDS Version 2.7).

**Figure S4.** Dose response of **3** in the EEEV-nLuc replicon assay

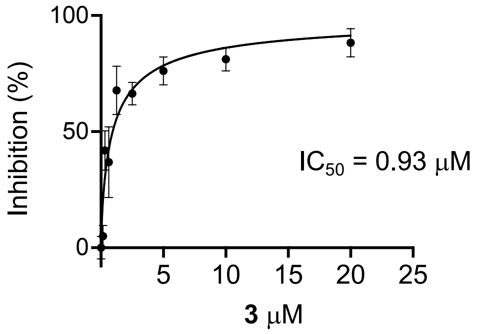


**Figure S5**. Cell toxicity data upon 48h exposure of **3** and **4**

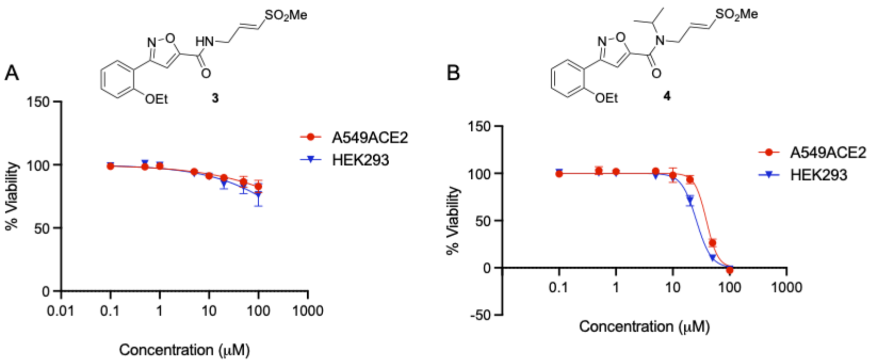


**Cell viability assay.** Cells were plated on a 384 well plate at a seeding density of 2000 cells/well for A549ACE2 (in DMEM high-glucose, 10% fetal bovine serum, 1X non-essential amino acids, and 2 mM L-glutamine) and HEK293 (in DMEM high-glucose, 10% fetal bovine serum) and allowed to adhere overnight. Cells were treated with dose response of compound, 1% DMSO (negative control), or 10% DMSO (positive control) for 48 h. CellTiter-Glo reagent (Promega) was added to each well and luminescence was detected on GloMax plate reader. There were two biological replicates containing treatments in quadruplicate. Cell viability was calculated using the following formula: ((raw RLU value – average of positive control wells) / (average of negative control wells – average of positive control wells))*100. Plots were generated using GraphPad Prism.

**Figure S6**. Site of covalent modification of CHIKV nsP2pro. MS/MS spectrum of the doubly charged ion (*m/z* 571.2441) corresponding to CHIKV nsP2 tryptic peptide ANVCWAK (aa 475–481). C478 was modified by a mass shift of +350.094 Da resulting from addition of SGC-NSP2PRO-1 (**3**). MS/MS spectra corresponding to a modified ANVCWAK (aa 475–481) peptide were observed in two independent samples (rep1 and rep2) treated with SGC-NSP2PRO-1 (**3**). The figure shows the MS/MS spectrum from rep2.

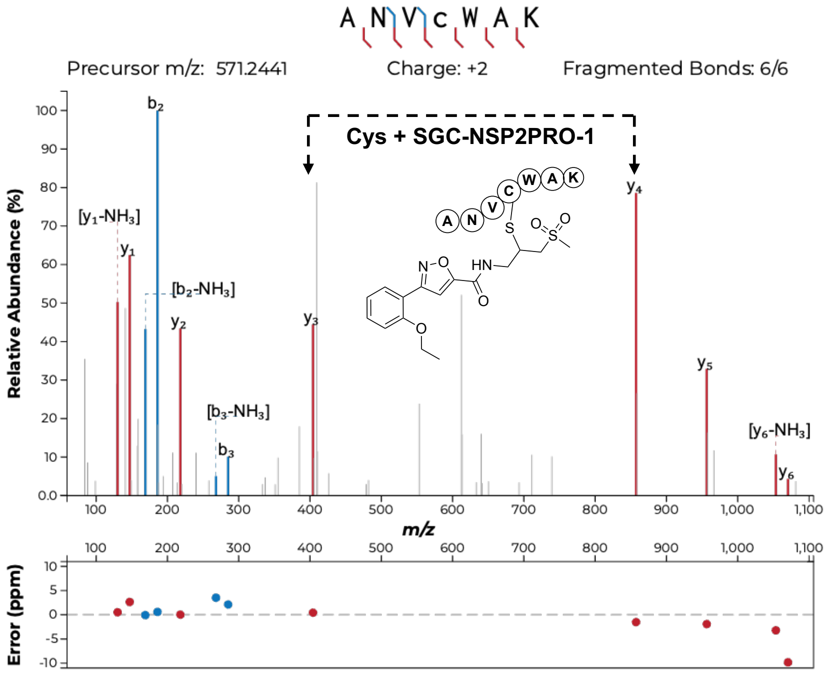

**Figure S7**. Dose-response curves of **VS**. A: Inhibition of CHIKV nsP2pro following a 30 min incubation. Values are the mean of triplicate determinations. B: Inhibition of CHIKV-nLuc replication in human fibroblast MRC5 cells at 6 h post-inoculation with the virus. Values are the average of duplicate determinations.

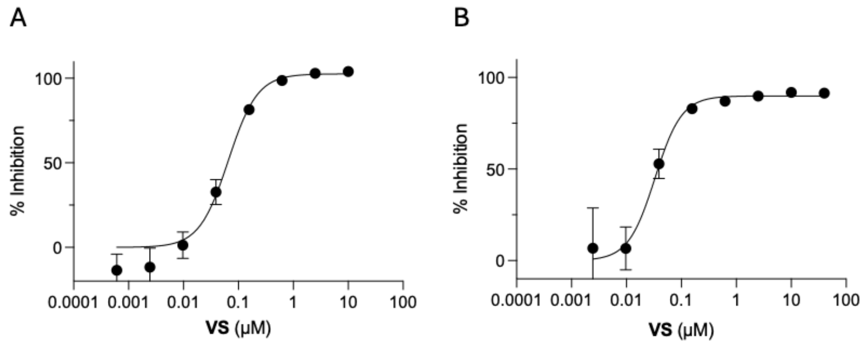

**Figure S8.** TAMRA fluorescence chemoproteomics of the vinyl sulfone warhead. **a.** Chemical structures of the simplified vinyl sulfone (**VSS**) and pyrazole chloroacetamide (**CA**). **b.** Uncropped fluorescent imaging of SDS-PAGE gel following covalent labeling of purified nsP2 (4 µg, lane P) by **TVS** or HEK293 cell lysates (140 µg total protein each, lanes 1 and 2) by **VSS** or **CA**. In lanes 1, **VSS** or **CA** were incubated at the indicated concentrations with cell lysates prior to click reaction to append the TAMRA fluorophore. In lanes 2 HEK293 cell lysates was supplemented with purified CHIKV nsP2. The expected M.Wt. of the nsP2 band is marked. No labelling of HEK293 cell lysate or nsP2 was observed with **VSS** (10 or 100 µM), while multiple proteins in the HEK293 cell lysate were weakly labelled by **CA** (10 µM). M lane contains molecular weight markers.

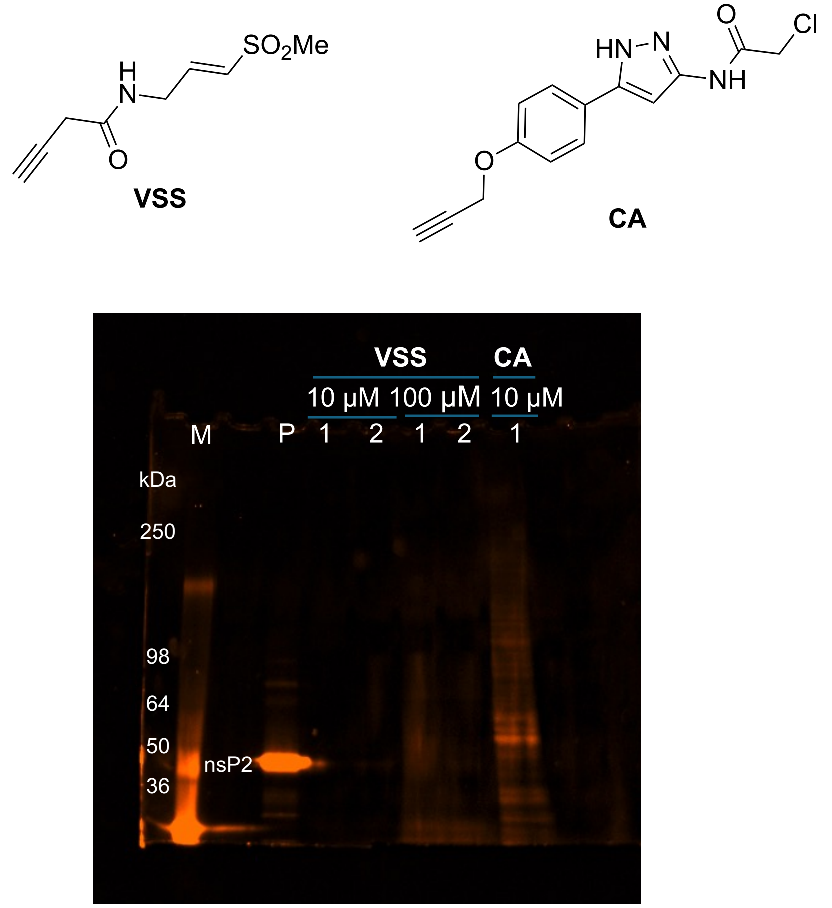

**Figure S9**. Structure of MOM-**1**

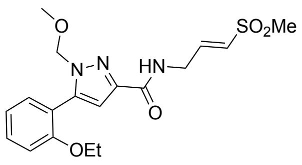

**Figure S10**. $^1$H NMR (400 MHz, DMSO-$d_6$) for SGC-NSP2PRO-1N (**4**)

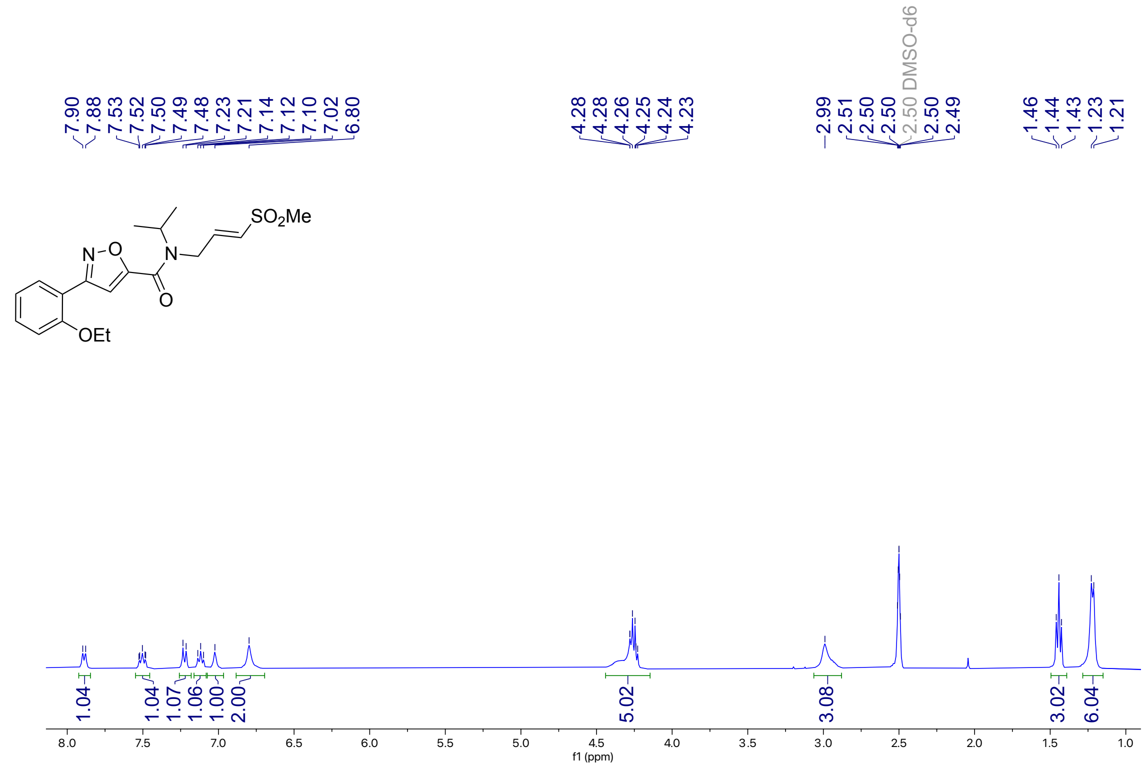


**Figure S11**. $^{13}$C NMR (126 MHz, DMSO-$d_6$) for SGC-NSP2PRO-1N (**4**)

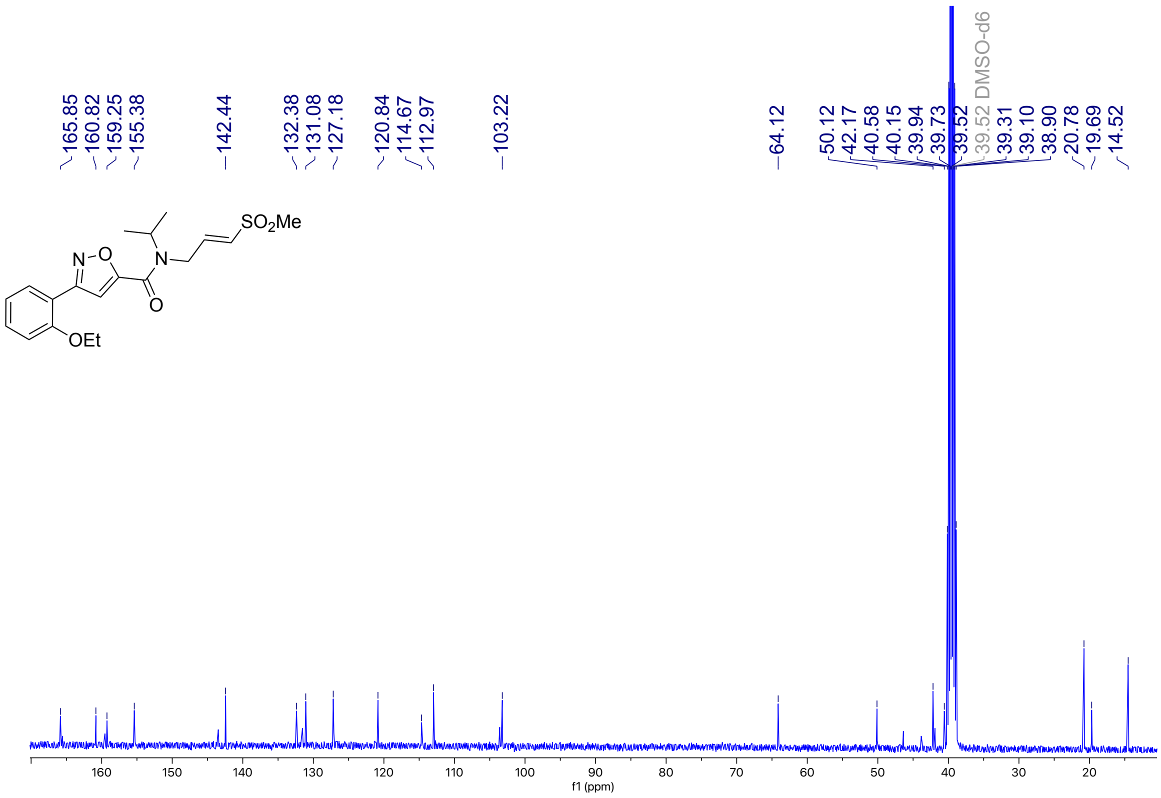

**Figure S12**. $^{1}$H NMR (400 MHz, DMSO-$d_6$) for **VS**

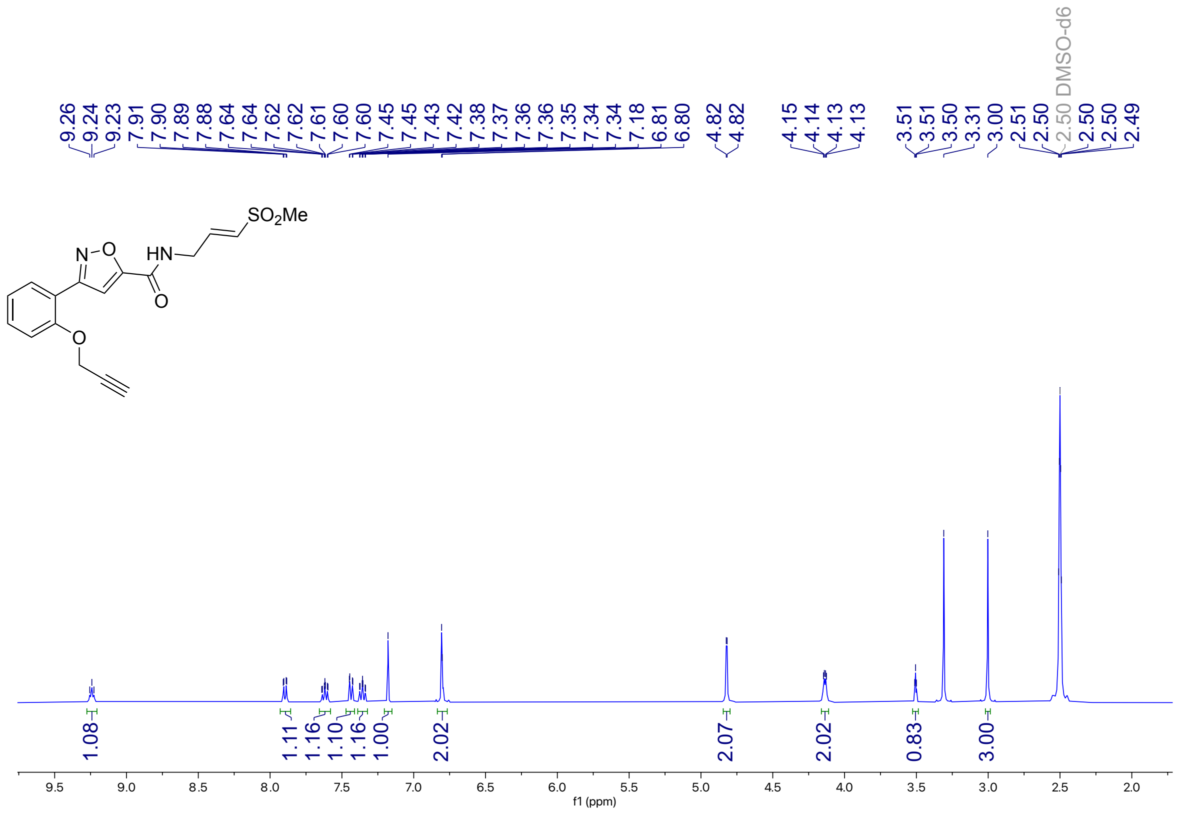

**Figure S13**. $^{13}$C NMR (101 MHz, DMSO-$d_6$) for **VS**

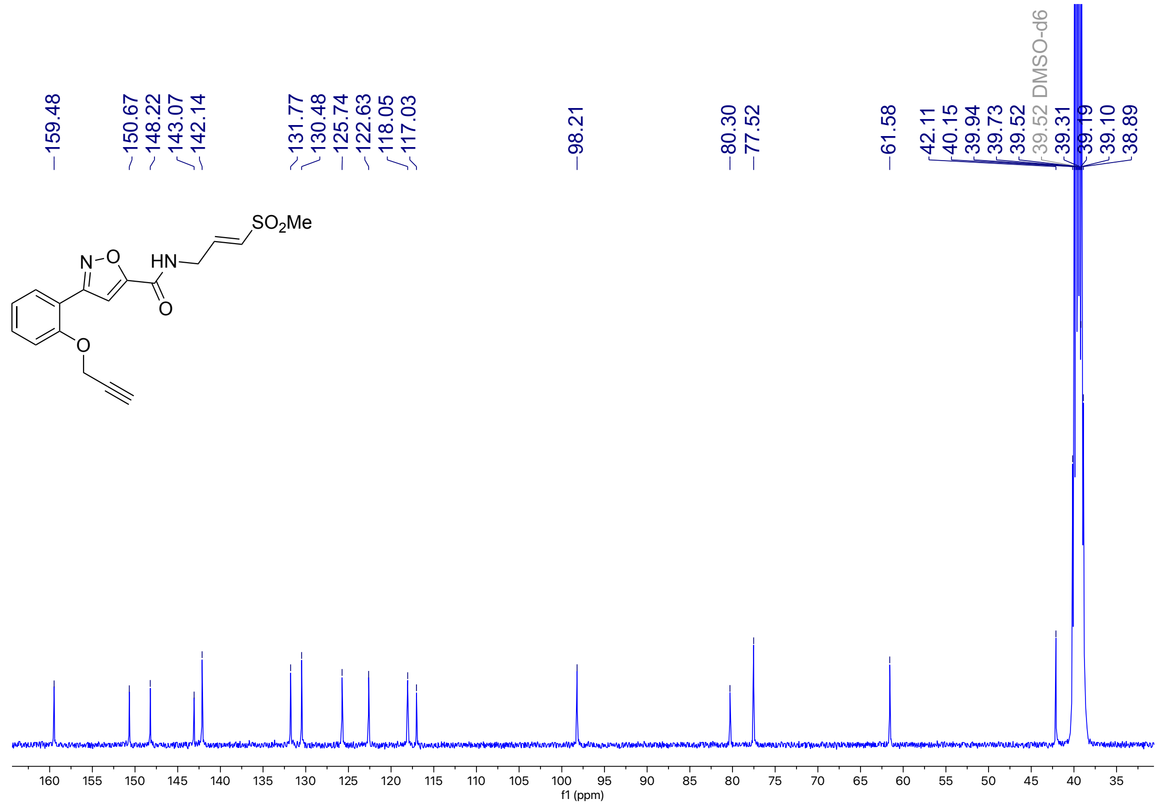

**Figure S14**. $^{1}H$ NMR (700 MHz, $CD_3CN$) for **TVS**

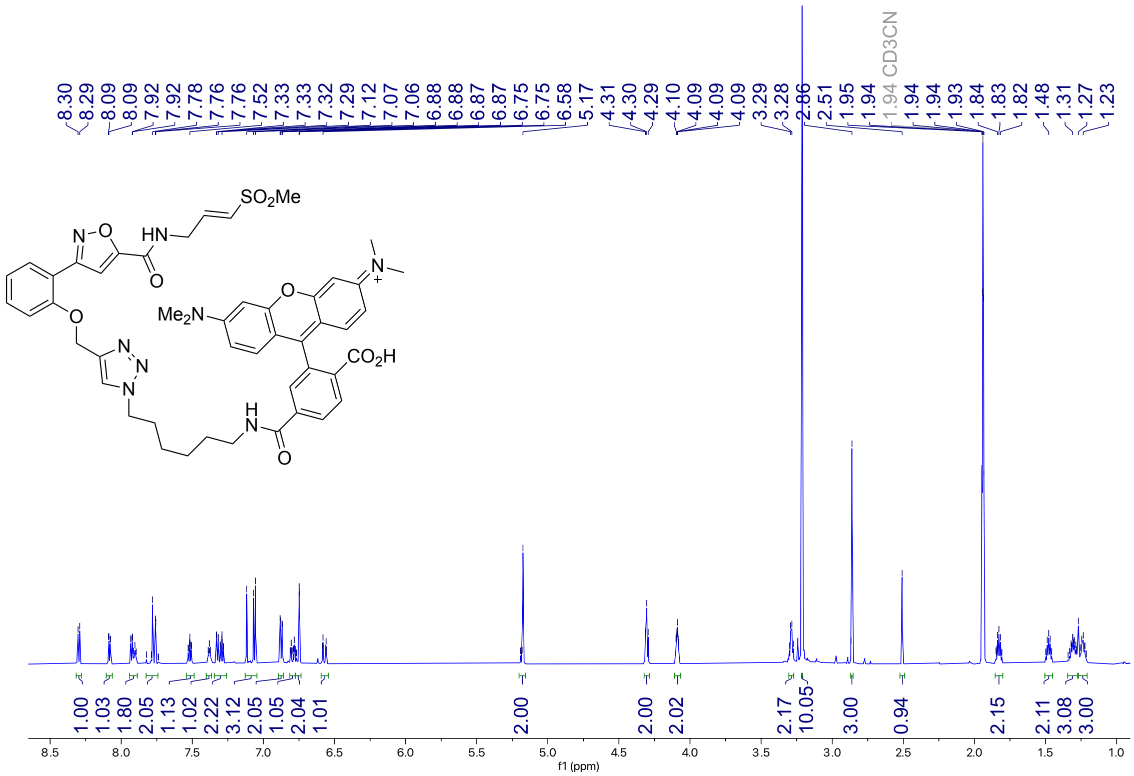


**Figure S15**. $^{13}C$ NMR (176 MHz, $CD_3CN$) for **TVS**

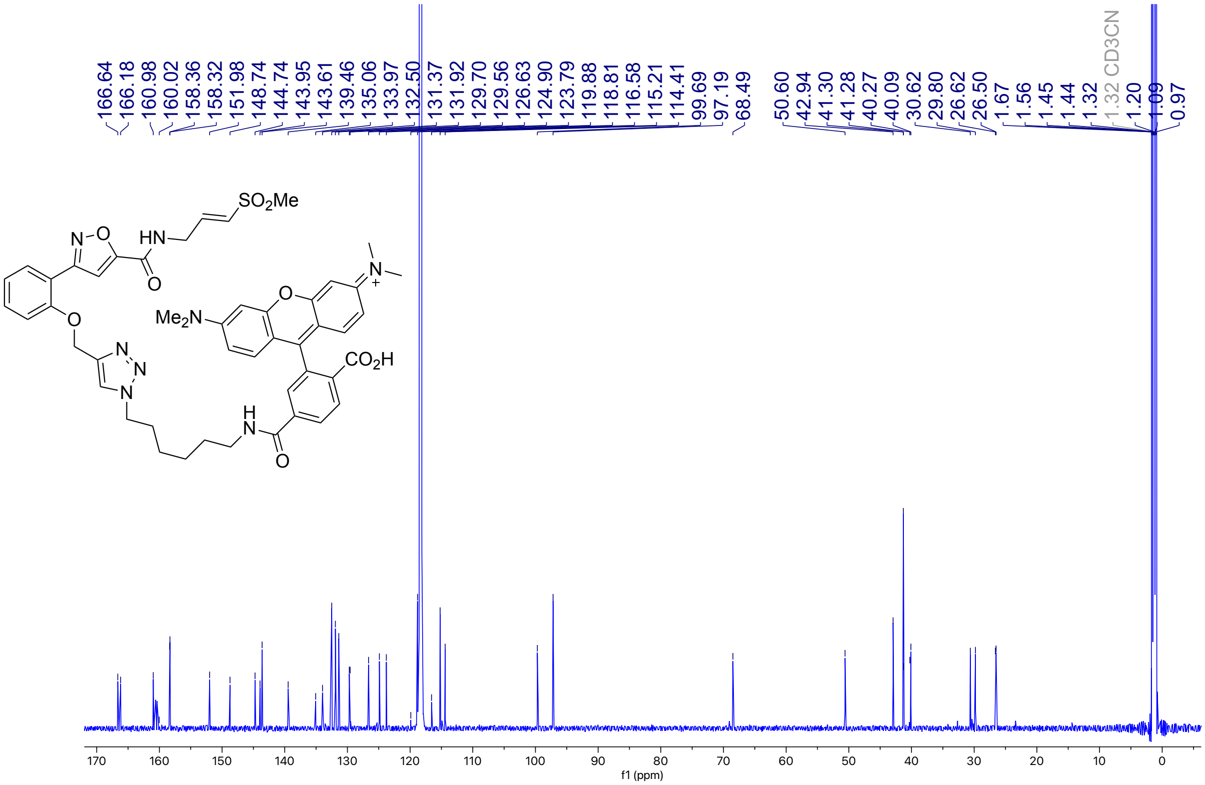

**Figure S16**. $^{1}$H NMR (400 MHz, DMSO-$d_6$) for **VSC**

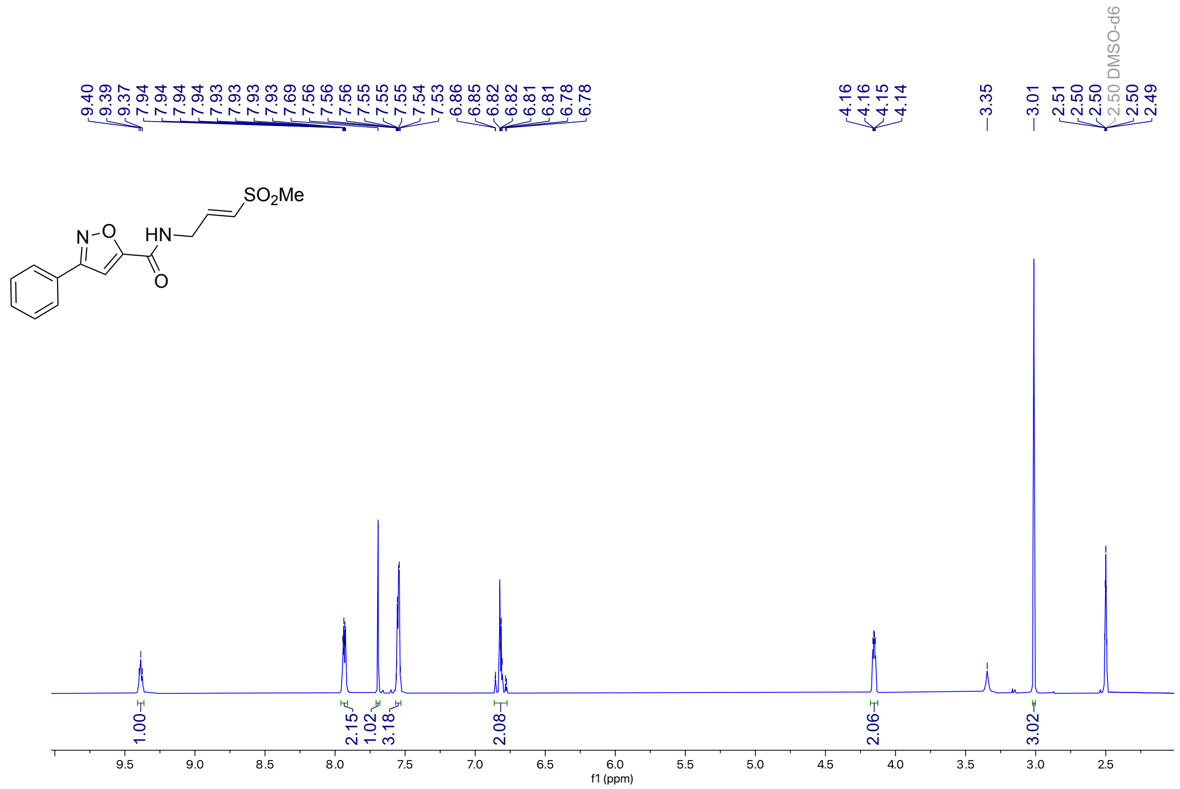


**Figure S17**. $^{13}$C NMR (101 MHz, DMSO-$d_6$) for **VSC**

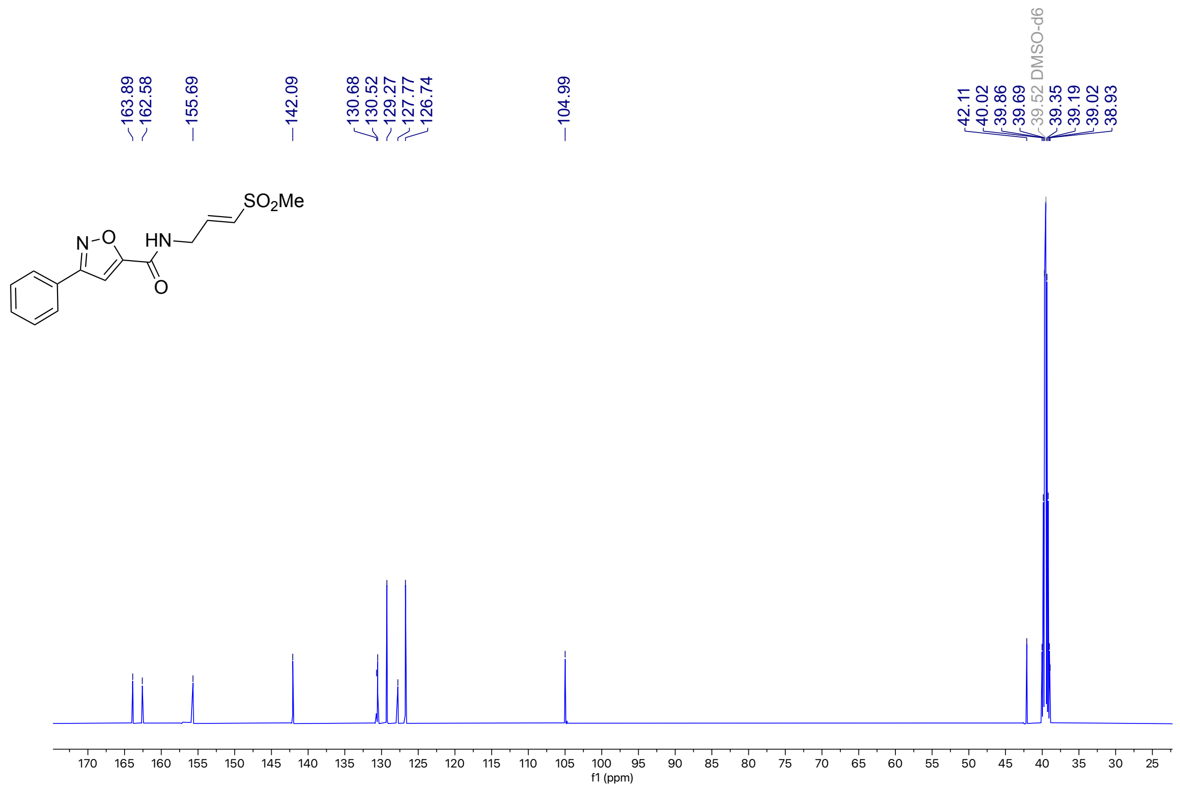

## HPLC analyses

**Figure S18**. HPLC trace of SGC-NSP2PRO-1N (**4**)

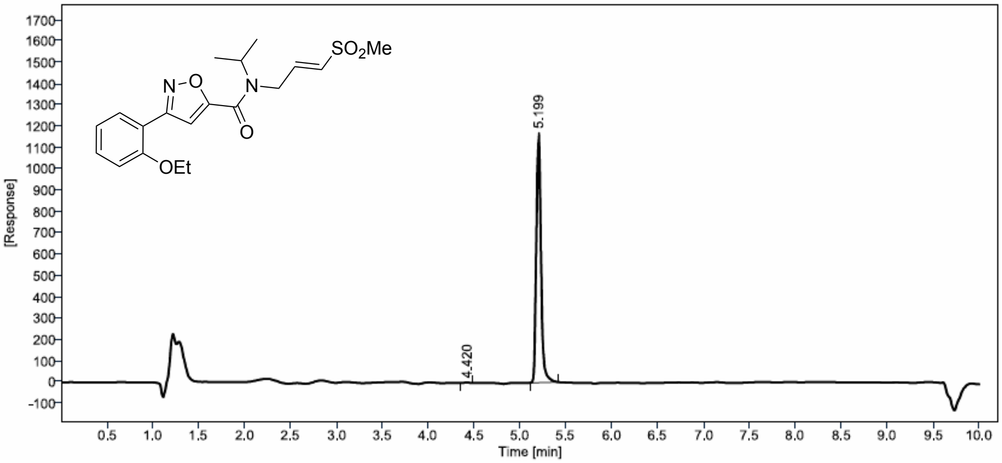


| Sr,No, | RT | Area | Height | % Area |
|---|---|---|---|---|
| 1 | 4,42 | 11 | 4 | 0,27 |
| 2 | 5,20 | 4174 | 1171 | 99,73 |

**Figure S19**. HPLC trace of **VS**

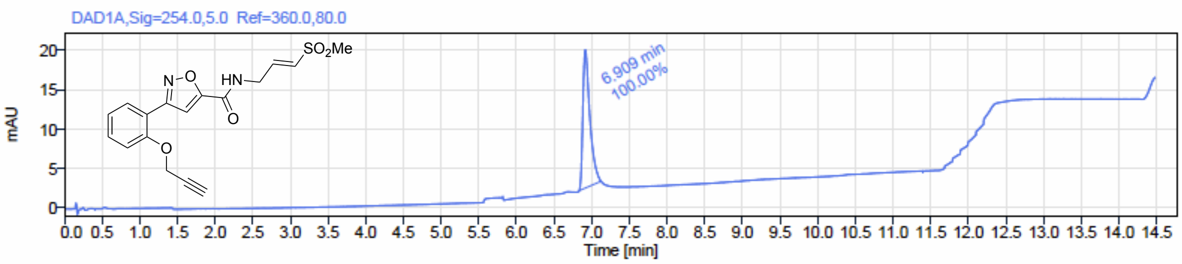


Signal Name DAD1A

| RT (min) | Signal description | m/z | Purity UV(‰) / MS(%) | Area | Area% |
|---|---|---|---|---|---|
| 6.909 | DAD1A,Sig=254.0,5.0 Ref=360.0,80.0 | | | 114.1 | 100.00 |

**Figure S20**. HPLC trace of **TVS**

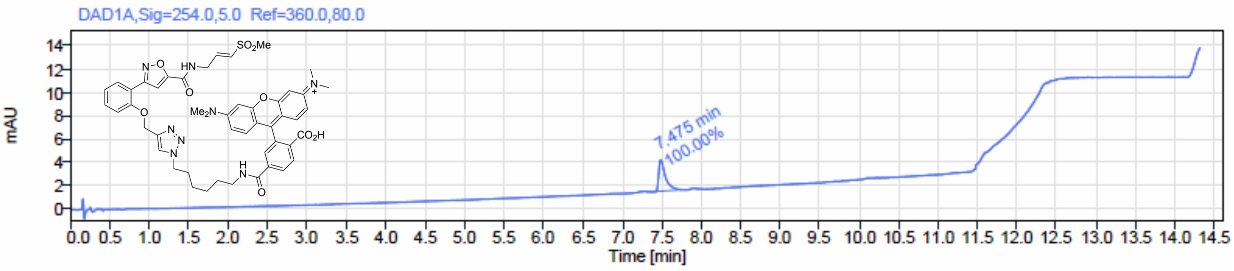


Signal Name DAD1A

| RT (min) | Signal description | m/z | Purity UV(‰) / MS(%) | Area | Area% |
|---|---|---|---|---|---|
| 7.475 | DAD1A,Sig=254.0,5.0 Ref=360.0,80.0 | | | 15.8 | 100.00 |

**Figure S21**. HPLC trace of **VSC**

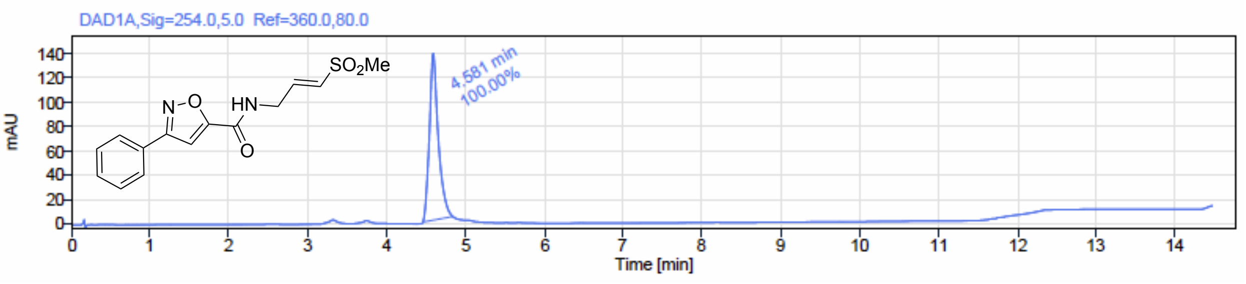


Signal Name DAD1A

| RT (min) | Signal description | m/z | Purity UV(‰) / MS(%) | Area | Area% |
|---|---|---|---|---|---|
| 4.581 | DAD1A,Sig=254.0,5.0 Ref=360.0,80.0 | | | 1079.2 | 100.00 |

**Figure S22.** Uncropped images of SDS-PAGE gels. **a.** Figure 4b. **b.** Figure 5a.

**a** Figure 4b

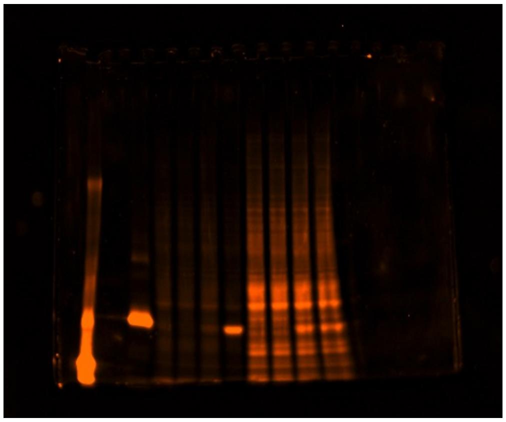

**b** Figure 5a

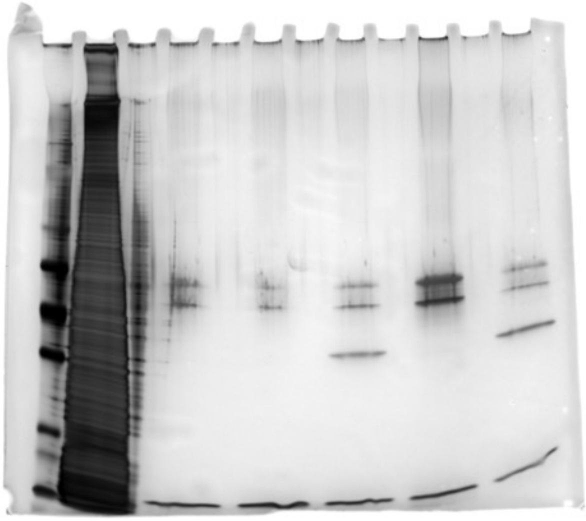

Supplement: Supplementary file 1 — Supplementary Information 1. [file 41598_2025_91673_MOESM1_ESM.pdf]
